# From skin to lung: basophil-mediated spread of allergic diseases

**DOI:** 10.3389/fimmu.2026.1916940

**Published:** 2026-09-07

**Authors:** E Da Choi, David Voehringer, Daniel Radtke

**Affiliations:** 1 Department of Infection Biology, Universitätsklinikum Erlangen and Friedrich-Alexander-Universität Erlangen-Nürnberg, Erlangen, Germany; 2 Friedrich-Alexander-Universität Erlangen-Nürnberg (FAU) Profile Center Immunomedicine, Erlangen, Germany

**Keywords:** allergic asthma, allergic comorbidities, atopic dermatitis, atopic march, basophils, lung, skin, type 2 inflammation

## Abstract

Basophils, the rarest type of granulocytes in peripheral blood, have emerged as important orchestrators of type 2 immune responses, including allergic inflammation. They were long considered merely as mast cell-related effector cells in IgE-mediated responses. However, they are increasingly recognized as critical orchestrators of type 2 immune responses and contributors to allergic disease pathogenesis. As significant producers of cytokines, chemokines, and eicosanoids, they are widely acknowledged as key modulators of dendritic cell (DC) function, innate lymphoid cell type 2 (ILC2) activity, and T helper 2 (Th2) cell differentiation, impacting both skin and lung inflammation. Human studies of atopic dermatitis (AD) have documented basophil infiltration within inflamed lesions, often concurrent with eosinophil recruitment, while murine models revealed a direct role in epidermal barrier dysfunction and chronic inflammation. Importantly, basophils exhibit context-dependent roles, also contributing to wound healing, highlighting the complexity of their function. In allergic asthma, they exacerbate responses by influencing memory Th2 cell migration, mucus production, and ILC2 activation. The long recognized epidemiological link between AD and allergic asthma underscores a critical gap in understanding how skin sensitization contributes to distal organ inflammation. Here, we discuss recent evidence establishing basophils as mediators of allergy, focusing on their role in sensitizing the lung via skin inflammation and exacerbating allergic lung disease. We highlight the mechanistic contribution of basophils to the comorbidity observed between skin and lung allergic diseases. Ultimately, understanding these mechanisms presents translational opportunities for therapeutic interventions aimed at preventing skin-mediated allergic sensitization and mitigating systemic allergic responses.

## Introduction

Basophils represent less than 1% of the leukocyte population in the blood (1). They show functional and morphological similarity to mast cells (MCs), which led to the early characterization of basophils as ‘blood circulating mast cells’ (2, 3). Once viewed primarily as blood-resident effector cells in IgE-mediated hypersensitivity, they are now also seen as modulators and effectors that infiltrate inflamed tissues and bridge innate and adaptive immunity (4–6). Upon activation by antigen-induced cross-linking of IgE bound to the high-affinity IgE receptor (FcεRI) or by tissue-derived alarmins such as IL-25, IL-33, and TSLP, basophils are capable of producing relevant amounts of IL-4, contributing to amplification of type 2 responses, especially in mice (7–9). However, epithelial alarmins do not act identically on basophils of humans and mice. While IL-33 activates basophils of both species, TSLP seems to activate basophils only in mice, and IL-25 had no effect on basophil IL-4 and IL-13 production in both humans and mice (10).

Increased numbers of basophils are observed in atopic dermatitis (AD) (11), a common chronic inflammatory skin disease with estimated prevalence rates up to 15-25% in children and 3-7% in adults worldwide (12, 13). Acute AD is characterized by a type 2 immunity-dominated immune response, skin barrier dysfunction, and pruritus. Later, type 2 immunity is still observed in chronic AD, which also often shows features of type 1 or type 3 immunity (14). Another prominent type 2 immunity-associated disease is allergic asthma, which is the most common chronic lung disease affecting up to 10% of children and 7% of adults worldwide. It is characterized by variable obstruction in the airflow, causing wheezing and dyspnea (15). According to the Gell and Coombs classification, both extrinsic AD, which occurs in up to 80% of patients (16, 17) and allergic asthma integrate characteristics of type I hypersensitivity reactions indicated by IgE-mediated sensitization with MC and basophil activation as well as delayed, cell-mediated type IV hypersensitivity reactions. These include type 2 responses with prominent contribution of type 2 cytokines, basophil and eosinophil recruitment, but also further promotion of IgE production by B cells (18–20). However, these classifications do not cover the full multifaceted nature of AD and asthma. Especially AD is a complex disease featuring barrier impairment and mixed immune responses (14). As a result of skin barrier damage, alarmins and cytokines can be released systemically and are sufficient to promote remote allergic sensitization in mouse models (21, 22). Of note, intrinsic AD is typically not associated with comorbidities or enhanced sensitization (16). In addition, cutaneous memory responses were described that fail to induce specific Th2 cells, LN responses or IgE (23), but their contribution to a spread of allergic diseases in the body remains elusive. Due to their relevance for sensitization and development of comorbidities, this review addresses primarily IgE-associated respiratory and cutaneous responses.

The epidemiological association between various IgE-associated (atopic) conditions is described in a framework called ‘atopic march’, which suggests frequent progression from early onset of AD to subsequent allergic asthma, allergic rhinitis, and food allergy, yet the underlying cellular and molecular mechanisms are incompletely understood (24).

**Figure 1:**
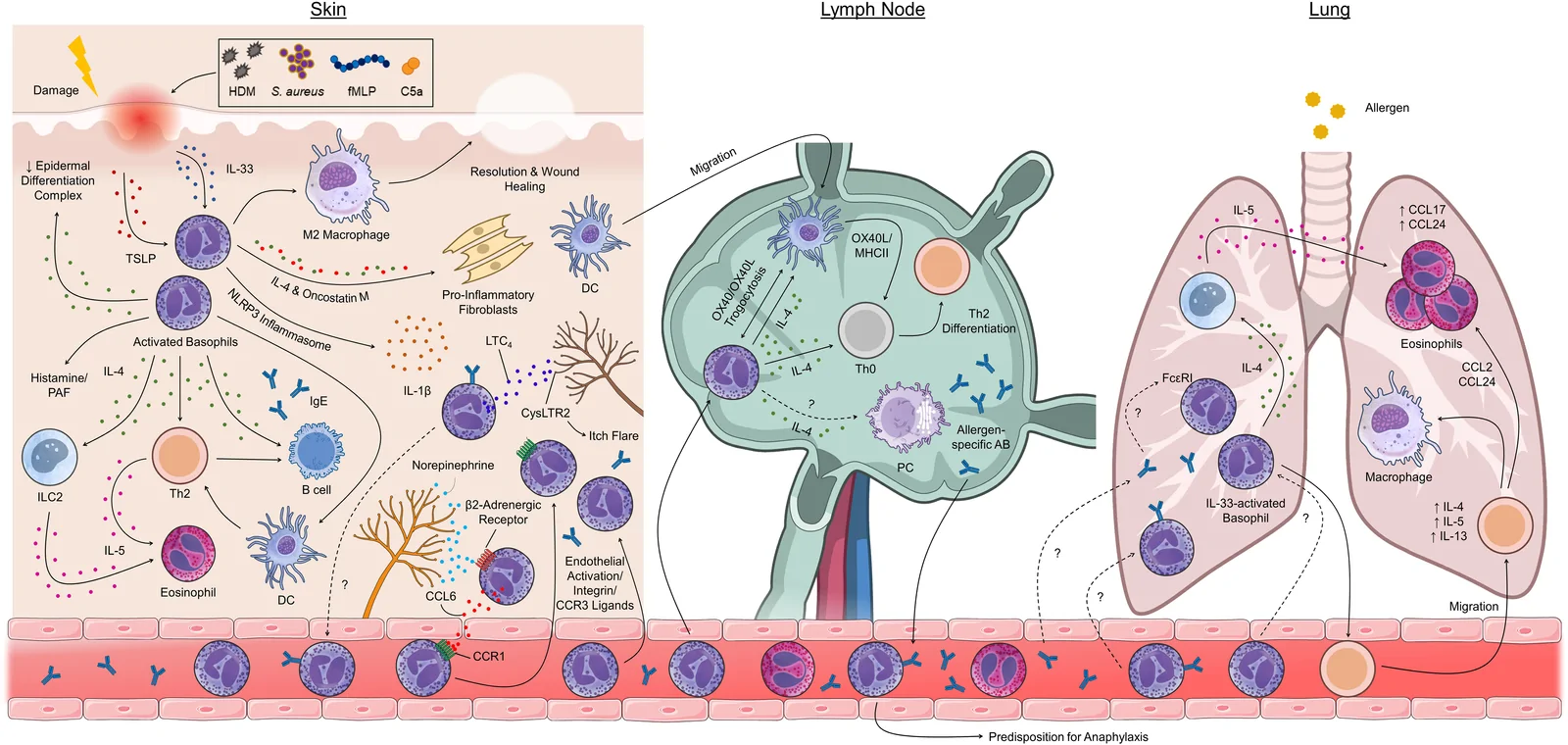
Immunopathological mechanisms of basophil-mediated type 2 inflammation from skin to lung via lymph nodes. (Left panel) Skin barrier disruption caused by exogenous stimuli (HDM, *S. aureus*, fMLP, C5a) or damage signals induces epithelial release of TSLP and IL-33, activating innate and adaptive immune cells. Activated basophils release histamine and platelet-activating factor (PAF), as well as IL-4, promoting Th2 cell and ILC2 activation and support B cells to become IgE producing cells, together with IL-5–driven eosinophil recruitment. Basophil-derived IL-4 and oncostatin M also contribute to a pro-inflammatory fibroblast activation circuit. In addition, basophil NLRP3 inflammasome activation leads to IL-1β release. Norepinephrine signaling via β2-adrenergic receptors induces CCL6 production, which binds CCR1 and promotes further basophil recruitment to inflamed skin. Allergen-stimulated basophils release leukotriene C4 (LTC_4_) and interact with sensory neurons; LTC_4_–CysLTR_2_ signaling contributes to acute itch responses. Basophils also contribute to resolution and tissue repair through M2 macrophage polarization. It remains unclear whether the IgE-loaded basophils migrate from the skin to the circulation and contribute to allergic inflammation elsewhere. (Central panel) In the draining lymph node, dendritic cells (DCs) migrated from the skin present allergens via MHC class II. Basophils and DCs interact through trogocytosis, OX40L–OX40 engagement, and IL-4/IL-4 receptor interaction, promoting differentiation of Th0 cells into Th2 cells. IL-4 appears to support plasma cell differentiation and IgE antibody production. (Right panel) In the lung, IL-33–activated basophils amplify type 2 cytokine responses, including IL-4 production. Recruited Th2 cells further enhance IL-4, IL-5, and IL-13 secretion. Together with other ILC2-derived signals, IL-5 promotes eosinophil recruitment and activation. This process is supported by chemokines such as CCL2, CCL24, and CCL17, as well as mediators derived from macrophages, leading to eosinophilic airway inflammation. It remains unclear if allergen-specific IgE-loaded basophils that migrate from the circulation into the lung and circulating allergen-specific IgE that binds to resident lung basophils are relevant to enhance allergen-specific responses.

This mini-review especially emphasizes the role of basophils in allergic skin and lung inflammation and combines information on how basophils might act as prominent mediators linking atopic conditions (**Figure 1**). We review functional characteristics of basophils, their role in experimental and clinical AD and allergic asthma, and discuss emerging evidence supporting basophils as contributors to allergic comorbidities and atopic march.

## Basophil biology and functional characteristics

Basophils originate from hematopoietic stem cells in the bone marrow. Mature basophils develop from basophil/mast cell progenitors or basophil progenitors, which both originate from granulocyte/monocyte progenitors (25). They circulate in blood and are recruited into inflamed tissues through endothelial activation, integrin-mediated adhesion (26), diapedesis, and migration along chemokine gradients, mostly composed of CCR3 ligands such as CCL11 (eotaxin-1) and CCL24 (eotaxin-2) (27), or CCL2 (28), and context-dependent CXCL12 (29). In addition, lipid mediators and cytokines can promote basophil recruitment into tissues. Notably, upon FcεRI-mediated activation, basophils can also upregulate additional chemokine receptors, including CCR4, CCR8, CCR9, CCX-CKR, CXCR4, and XCR1, potentially increasing their capacity for tissue homing during allergic inflammation (30). Although IL-33 does not directly attract basophils, it primes them in the presence of IL-3. This leads to stronger adhesion, migration toward CCL11, and robust cytokine release, thereby amplifying type 2 inflammation in tissues (31).

Basophil activation can occur through multiple pathways. Cross-linking of FcεRI-bound IgE by allergen (32), direct stimulation by cytokines such as TSLP, IL-33 (often in combination with IL-3), by the microbial peptide fMLP (33), or through engagement of the anaphylatoxin C5a complement receptor (34, 35). Activation of basophils leads to the rapid release of histamine granules (32), leukotriene C4 (36), and platelet-activating factor (PAF) (37), and secretion of IL-4 to sustain and amplify Th2 immunity (38). Basophil-derived IL-4 promotes B cell class switching to IgE, supports Th2 differentiation of naïve CD4^+^ T cells, and can also promote ILC2 proliferation (39, 40). Additionally, basophils can directly interact with dendritic cells (DCs) to modulate antigen presentation and Th2 priming (41). Recent evidence further highlights their cross-talk with fibroblasts, establishing a pro-inflammatory stromal axis in allergic tissues driven by basophil-derived IL-4 (42).

## Basophils in allergic skin disease

Basophils are consistently detected in lesional skin of AD patients, where their numbers correlate with disease severity and Th2 cytokine levels (11). Fundamental work demonstrated that basophils orchestrate chronic AD in a mouse model of hapten-induced skin inflammation, with basophil depletion substantially reducing disease severity (3, 43). Later studies showed that skin-infiltrating basophils are major producers of IL-4 in the inflamed skin dermis (44) and that their presence correlates with eosinophil recruitment, showing a relationship that is mediated by basophil-dependent eotaxin release (45).

Studies using multiple AD-relevant mouse models have demonstrated functions of basophils in skin inflammation. In MC903 (calcipotriol)-induced AD, basophils infiltrate the skin and drive the production of Th2 cytokines (46, 47). In filaggrin-deficient models, in which AD is caused in response to barrier impairment (48), basophil accumulation enhances the production of TSLP and amplifies sensitization and inflammation (49). In house dust mite (HDM) and ovalbumin (OVA) epicutaneous sensitization models, as well as *Staphylococcus aureus*-induced skin inflammation (mediated via IL-33 signaling), basophils have been shown to be essential for the induction and maintenance of AD-like pathology (50, 51). Strikingly, in the *S. aureus* model, IL-33 primes basophils to activate the NLRP3 inflammasome and produce IL-1β, a cytokine that is also elevated in patients with AD and correlates with disease activity (52). These findings reveal an additional inflammasome-dependent mechanism by which basophils contribute to skin inflammation (50).

TSLP, released by keratinocytes in response to barrier damage, allergens, or microbial stimuli, potently activates basophils via TSLP receptor signaling, promoting their IL-4 release and enhancing FcεRI expression. TSLP-activated basophils are sufficient to drive Th2-skewed immune responses *in vivo*, supporting their non-redundant role in allergic disease models such as AD and allergic asthma (53). However, it has also been shown that mice with TSLP-R-deficient basophils mount a normal ear swelling response to MC903, indicating that TSLP signaling in other cell types is more relevant in this AD model (54). IL-33, similarly released by damaged epithelium and dermal stromal cells, acts on basophils to enhance IL-4 production and IgE-mediated activation. TSLP, IL-33, and IL-4 cytokine signals link the barrier-defective microenvironment of AD skin to basophil activation with consequences that extend beyond the skin itself (10, 20).

It has been demonstrated that basophils not only promote initial skin barrier disruption but also participate in the resolution phase of atopic skin, and furthermore show promotion of wound healing in adult and aged mice, suggesting a dual role that may vary with disease stage (55, 56). Furthermore, it has been shown that the skin barrier function is regulated by a balance between the aryl hydrocarbon receptor and IL-13/IL-4-JAK-STAT6/STAT3 axis, which also promotes oxidative stress and inflammation (57). More recently, single-cell and spatial transcriptomic analysis of mouse models have identified a basophil-fibroblast pro-inflammatory circuit, which is driven by basophil-derived IL-4 and oncostatin M. This pro-inflammatory circuit amplifies type 2 skin inflammation and can be therapeutically disrupted by targeting fibroblast IL-4Rα and gp130 pathways (42). This finding underscores that basophils’ effects extend beyond the immune compartment to directly remodel the stromal microenvironment.

## Basophils in allergic lung inflammation

Basophils are recruited to the lungs during airway inflammation and accumulate in bronchoalveolar lavage fluid (BALF) and lung tissue of both asthmatic patients and corresponding mouse models. They are described to amplify DC-induced Th2 and memory Th2 cell responses that drive established allergic asthma in mice and humans (51, 58, 59). However, the role of basophils in lung inflammation remains complex and context-dependent. Several studies have reported a lack of early or discernible effects of basophils in lung inflammation (17, 43, 60), yet others highlight their significant contributions. As such, the induction of primary Th2 responses to inhaled house dust mite (HDM) allergen appears largely driven by inflammatory DCs rather than basophils (17). On the other hand, recent research has elucidated a mechanistic role for basophils in allergic asthma, demonstrating that IL-33-activated basophils promote disease progression by regulating Th2 cell entry into lung tissue. Basophil depletion in this model impaired Th2 cell recruitment and reduced hallmarks of allergic asthma, including goblet cell hyperplasia and mucus overproduction (61). Further, basophils are increasingly recognized for their ability to shape lung inflammation through mechanisms such as IL-4-mediated regulation of ILC2s, a process promoting eosinophilic airway inflammation (62). In murine eosinophilic allergic asthma, basophils appear to influence local lung eosinophil populations, facilitating their recruitment and survival, with basophil depletion demonstrably reducing pulmonary eosinophilia and inflammatory cell numbers (63). These findings collectively support the concept that basophils are not simply effector cells, but rather active regulators of immune responses within the lung, although their precise role may vary depending on the specific inflammatory context.

## Basophils as a bridge between skin and lung: insights into the development of allergic comorbidities

A recent study has shown that murine basophils contribute to systemic antigen sensitization through the skin by induction of allergen-specific antibody that functionally primed spleen cells (51). It has further been demonstrated that TSLP-activated basophils are critical promoters of subsequent lung inflammation in a mouse atopic march model. Here, skin-derived TSLP-activated basophils amplified lung inflammatory responses upon allergen challenge, providing evidence for a TSLP-basophil-driven link between both organs. It showed that epicutaneous sensitization increased basophil activation and IL-4 production, leading to eosinophilic lung inflammation. Depletion of basophils significantly reduced airway inflammation, demonstrating their central pathogenic role. The study further identified TSLP as a key upstream driver, since elevated TSLP levels in inflamed skin promoted basophil activation, while TSLP-R-deficient mice exhibited attenuated lung inflammation and reduced type 2 immune responses (64). Experiments with genetically basophil-deficient mice and mice in which basophils were temporarily depleted during sensitization have shown that basophils were required during skin sensitization for promoting subsequent eosinophilic lung inflammation in airway-challenged mice (65). Mechanistically relevant, basophils enhanced the expression of CCL24, a potent chemokine for eosinophil recruitment to the lung, and CCL17, associated with Th2 expansion and recruitment (65). This study separates the role of basophils in skin‐mediated sensitization from their downstream effector functions to understand how basophils promote predisposition to allergic multimorbidity, providing evidence for a causal role of basophils in distal organ sensitization. As a limitation, replenishing basophils after temporary depletion might not reflect the activation state of basophils already present during sensitization. Interestingly, transcription of IL-4 and IL-13 in the challenged lung was only reduced when basophils were absent during lung inflammation, suggesting that lung-recruited basophils rather than basophils present during skin-mediated sensitization caused upregulation of IL-4 and IL-13 expression in the lung (65).

The mechanisms by which skin-sensitized basophils promote lung inflammation likely involve multiple pathways. Basophil-dependent enhancement of IgE production increases the reactivity of lung-resident MCs to aeroallergens, creating an IgE-mediated amplification loop initiated in the skin (60, 66). Alternatively, systemically elevated basophil-derived factors like IL-4 and IL-13 may condition the lung microenvironment to enhance Th2 cell and ILC2 responses/migration independently of antibody dissemination. Another possibility is that basophils or other cells activated in the skin may upregulate homing receptors that facilitate their recruitment to the lung upon subsequent allergen exposure.

Furthermore, emerging evidence suggests that basophils contribute not only to distal lung sensitization but also to allergic inflammation at other barrier sites. In particular, basophils are known to contribute to the skin–gut axis, linking cutaneous sensitization to systemic food allergic responses. In a recent study, it has been demonstrated that *Staphylococcus aureus* exposure during cutaneous antigen sensitization promotes basophil- and IL-4-dependent exacerbation of food anaphylaxis in mice. Mechanistically, basophil-derived IL-4 enhances DC-mediated Th2 polarization, thereby amplifying systemic allergic responses to food antigens (67). Beyond their cytokine-producing capacity, basophils may further modulate adaptive immune responses through direct interactions with DCs. Activated basophils have been shown to acquire antigen-presenting and costimulatory molecules from DCs via trogocytosis (41) and to upregulate OX40L expression, enabling them to promote Th2 differentiation and allergic inflammation. Notably, these mechanisms have also been implicated in eosinophilic airway inflammation (68), suggesting that basophils function as important amplifiers of type 2 immunity across multiple barrier tissues. Collectively, these findings support a broader role for basophils in coordinating inter-organ allergic responses, including the modulation of both the skin–gut and skin–lung axes.

## Clinical and translational implications

The evidence reviewed here positions basophils as mechanistically important contributors to atopic comorbidities and atopic march that link skin and lung inflammation. Early identification of the severity and extent of atopic sensitization is important for predicting the progression of AD to allergic asthma and other atopic diseases. As IgE-mediated activation of basophils plays a central role in atopic disease (69), the basophil activation test (BAT), a flow cytometric assay that measures allergen-induced upregulation of CD63 and CD203c on basophils, has emerged as a sensitive tool for detecting IgE-mediated sensitization (70–72). It may help predict atopic disease progression in the future, but further longitudinal studies are needed to support this hypothesis. There are several approved biologics that directly and indirectly target basophil biology. Dupilumab, an anti-IL-4Rα antibody, suppresses IL-4 and IL-13 mediated signaling. This inhibits both basophil effector functions and the downstream Th2 responses promoted by basophils (73). Omalizumab, an IgE-sequestering antibody, reduced the surface FcϵRI expression on basophils, which dampens the activation via surface-bound IgE (74). Tezepelumab, a TSLP-neutralizing antibody, can interrupt an important upstream alarmin signal, which can activate basophils in the skin and the lung (75). Beyond biologics, but only explored in mice, topical application of a PDE4 inhibitor was described to ameliorate AD-like inflammation through inhibition of basophil IL-4 production (76).

Clinically, dupilumab has demonstrated benefit in patients with AD (73) and a reduction in allergic asthma exacerbations (77). Omalizumab is associated with significant reductions in asthma exacerbation rates (78), however, its efficacy in AD remains less well-established, with variable findings across studies (74). Tezepelumab has demonstrated efficacy in patients with severe asthma, and baseline blood basophil levels are being explored as a potential predictive biomarker for treatment response (75). Nevertheless, early identification of susceptible individuals to interrupt basophil-mediated dissemination of allergic sensitization to prevent or attenuate atopic march remains a challenge and needs further investigation.

Early treatment of skin disease may provide a critical opportunity to prevent basophil-driven allergic sensitization beyond the skin to distal organs throughout the body. If basophils that are activated in inflamed skin directly contribute to later allergic inflammation in the lungs, then early management of AD could offer benefits that extend beyond improving skin symptoms alone. In particular, therapies that restore the skin barrier, reduce inflammation, and limit allergen exposure during infancy may help interrupt the atopic march and decrease the risk of developing allergic asthma later in childhood. This highlights the importance of controlling skin inflammation during early disease development, when allergic pathways might imprint long-term outcomes. Future clinical studies should consider basophil-related biomarkers to clarify the mechanisms linking AD and allergic asthma and determine whether early intervention can meaningfully reduce the development of allergic asthma and other allergic diseases later in life.

## Future directions

Despite significant progress in understanding basophils in allergy, important questions remain. The relative contribution of basophils to human allergic diseases, compared to the robust evidence from murine models, requires further attention. Human basophils in lesional AD skin are present at lower frequencies than in mice (79, 80), and their precise functional state *in vivo* remains incompletely characterized. Single-cell RNA sequencing studies in human atopic skin and BALF have begun to reveal basophil transcriptional heterogeneity (81), but the functional significance of distinct basophil subsets in disease pathogenesis is unknown. Determining whether circulating and tissue-infiltrating basophils represent distinct subpopulations with different pro-inflammatory or immunomodulatory capacities will be important for both mechanistic understanding and for identifying the most therapeutically relevant targets.

The precise route through which skin-sensitized basophils promote signals to the lung is also unclear. Whether basophils physically migrate from skin to lung, whether the systemic release of soluble factors from inflamed skin tissues contributes to distal immune conditioning, or whether skin-derived mediators condition the circulating bone marrow-derived basophil progenitors to adopt a lung-homing phenotype, remains to be determined. Additionally, the interactions between basophils and endothelial cells may represent a potential mechanism underlying distal organ sensitization in the context of atopic march.

Previous studies have shown that basophils orchestrate the recruitment of eosinophils during IgE-dependent dermatitis through interactions with endothelium and the production of IL-4. IL-4 upregulates endothelial VCAM-1 expression, which facilitates eosinophil adhesion and accumulation within inflamed tissues (82). These findings suggest that basophils may directly modulate endothelial activation and vascular permeability, thereby promoting inflammatory cell infiltration likely relevant for subsequent systemic sensitization and progression of atopic diseases beyond the skin.

In addition to immune-cell interactions, neuroimmune crosstalk has emerged as a critical regulator of allergic inflammation in skin barrier tissues (83, 84). It has been observed that upon allergen challenge with MC903 and OVA allergen, murine basophils migrate into the skin and are observed consistently in close proximity to sensory nerve fibers. Allergen-stimulated basophils release leukotriene C4 (LTC_4_), which engages with CysLTR2-expressing sensory neurons to mediate acute itch flares (85). Furthermore, it was shown that sympathetic released norepinephrine can bind adrenergic receptor on basophils inducing CCL6 chemokine release. CCL6 further drives basophil recruitment and exacerbates skin inflammation (86). Recently, it has been shown that neuronal substance P activates MrgprX2 receptor on MCs, while MC-derived mediators such as tryptase and IL-31 enhance neuronal excitability and neuropeptide release. These signals can form a local amplification loop that is further strengthened in chronic inflammation by increased nerve growth, higher synaptic density, and narrowing of the synaptic cleft (87). Given the functional similarity of basophils with MCs, the findings in MCs encourage further investigation of the role of basophils in neuro-immune crosstalk and AD.

Finally, the role of basophils in other atopic inter-organ responses, such as their contribution in the skin-gut axis to promote food allergy, suggests that basophil-mediated distal organ priming may be a general principle rather than a skin-lung specific observation (67). A broader understanding of basophils as cells linking local inflammation and sensitization to distal organ responses has the potential to inform novel diagnostic and therapeutic strategies across atopic diseases, underscoring a need for further research.
